# Should Internal Mammary Lymph Node Biopsy be A Routine Step in Recurrent Breast Cancer? Report of Three Cases With Negative Axilla and Positive Internal Mammary Node

**DOI:** 10.4021/wjon391w

**Published:** 2011-12-19

**Authors:** Stavros E Chatzopoulos, Kasim A Behranwala, Parveen Jayia, Ragheed Al-Mufti R, Susan J Cleator, Dimitri J Hadjiminas

**Affiliations:** a Imperial College Healthcare NHS Trust, St Mary’s hospital, London W21NY, UK

**Keywords:** Breast cancer, Loco-regional recurrence, Internal mammary lymph node biopsy

## Abstract

Lymph node status is the most important clinicopathological prognostic factor for breast cancer patients and in most breast units it reflects only the axillary lymph nodes. A second often overlooked basin consists of the internal mammary lymph nodes (IMLNs) whose evaluation is not done as a routine step during the staging process. We highlight the need to consider incorporation of IMLNs into a patient’s staging by presenting three cases of recurrent breast cancer with negative axilla and positive IMLN, a finding which altered their final management. We suggest that biopsy of IMLN should be a routine step in recurrent breast cancer when axillary lymphatics are disrupted by previous surgery although further research is required to define the optimal management of node positive cases.

## Introduction

The lymph node status is the most important clinicopathological prognostic factor for breast cancer patients. The axillary lymph nodes are the main lymph node basin of the breast, hence the main determinant of the patient’s lymph node status. A second often overlooked basin consists of the internal mammary lymph nodes (IMLNs) [1], whose evaluation (imaging or operative) is not done as a routine step during the staging process in most breast units. An alternative lymphatic drainage is possible in patients with local recurrence, who have been previously managed with axillary dissection or axillary sentinel lymph node biopsy (SLNB) [2]. In the setting of local recurrence, the proper evaluation of the IMLN might be more significant since they may be rendered the main lymphatic basin following disruption of the axillary lymphatics by previous surgery.

SLNB is the “gold standard” for primary breast cancer patients and has been validated by numerous studies showing high sensitivity and specificity in detecting axillary and IMLN involvement [3, 4]. The false negative rate of the SLN is reported to be between 5% and 10%. Axillary lymph node dissection is mostly done if the SLNB is positive. The role and effectiveness of SLNB in patients with locally recurrent breast cancer is not clear.

Detection of involvement of IMLNs in breast cancer patients and their implications for management constitutes a growing topic in the literature. The involvement of IMLNs is frequently underestimated and patients may be undertreated. We present three cases of locally recurrent breast carcinoma illustrating how previous surgical intervention in a patient’s axilla may render the IMLNs the primary lymphatic basin for the whole breast.

## Case Report

### Case 1

A 39 years old woman underwent quadrantectomy with SLNB (0/2) for a multifocal, grade 2, oestrogen receptor (ER) progesterone receptor (PR) positive and HER2 negative invasive lobular carcinoma of the right breast at 9 o’clock position in 2006. Post-operatively, she received adjuvant radiotherapy to the remainder of her right breast followed by tamoxifen and a GnRH agonist. Four years later, she presented with local recurrence in the same upper outer quadrant of her right breast. Staging investigations with CT and bone scintigraphy showed no metastatic disease. Lymphoscintigraphy, performed with scans at 0.5, 1.2 and 4 hours post-injection of 32 mBq of Tc-labelled nanocol, did not reveal any focal accumulation of radioisotope. She underwent completion mastectomy with axillary clearance and biopsy of the 3rd intercostal space IMLN. Histological examination revealed invasive lobular carcinoma with lymphovascular invasion and 27 negative axillary lymph nodes. The only positive node was the 3rd intercostal space IMLN. Adjuvant chemotherapy was given followed by aromatase inhibitor and GnRH agonist.

### Case 2

A 52 years old woman was treated by microdochectomy for single duct nipple discharge of her right breast followed by wide local excision and SLNB for high grade DCIS approximately 20 mm in maximum diameter, with micropapillary pattern and tumour free margins of greater than 10 mm, in 2003. There were four negative SLN’s retrieved. No adjuvant treatment was given. A surveillance mammogram, two years later, showed a tumour in the upper-inner quadrant of the same breast. Staging with CT and bone scintigraphy was negative. She underwent wide local excision with axillary lymph node sampling following a failed attempt to identify the sentinel node. The 2nd intercostal space IMLN was also biopsied. Histopathological examination revealed a 20 mm, grade 2, ER positive and HER2 negative invasive ductal carcinoma with metastasis in the IMLN. The axillary node sample was negative and there was no lymphovascular invasion. The patient was treated with adjuvant chemotherapy (FEC_60_ X 6 cycles), radiotherapy to the remainder of her right breast and anastrozole for 5 years.

### Case 3

A 68 years old patient presented with right breast lump of the lower outer quadrant. She had undergone right axillary clearance for metastatic adenocarcinoma in the right axilla 20 years previously. This could have been an occult breast primary but details were unclear. She had not received any treatment to the right breast at the time. On this occasion her breast cancer was confirmed by pre-operative core biopsy. Lymphoscintigraphy showed a hot spot in level 3 of her ipsilateral axilla indicating that this may not have been dissected previously. She underwent wide local excision, axillary clearance of the third level corresponding to the area detected by the lymphoscintigraphy and IMLN biopsy of the 3rd intercostal space. Histopathology showed a 25 mm, grade 3 invasive ductal carcinoma, lymphovascular invasion, negative level III axillary nodes (0/5) and positive IMLN. ER and PR are both positive. She was also advised to have adjuvant radiotherapy (including the breast and the IMLNs), chemotherapy and an aromatase inhibitor for 5 years.

## Discussion

The feasibility of SLNB in patients with recurrent disease has been demonstrated by many studies, with identification rates varying between 55 - 100% [5]. A common observation of these studies is the high incidence of non-axillary locations of the SLN. Port E et al who reported non-axillary drainage in only 6% for primary breast cancer operations [6], showed for the rate to be 30% in the subset of patients having operations for recurrence. In another series of regional recurrences, the incidence of IMLN involvement was 18% (7 of 39). In the latter report 3 patients had isolated IMLN metastasis only whereas a further 4 patients had IMLN metastases as well as involvement of lymph nodes at other sites [7]. Similar findings have been observed in other series with drainage away from the ipsilateral axilla reported in 30 - 78% of cases [5]. While the role of routine lymphoscintigraphy in primary SLNB remains a matter for debate, its role is central in the re-operative setting because of altered lymphatic drainage by previous surgery. However, 2 of our cases demonstrate that lymphoscintigraphy is not necessarily helpful.

The biopsy of the IMLN was based on the surgeon’s judgement according to the tumour location. Lymph draining from the upper half of the breast flows to the first and second IMLN while lymphatics originating in the lower half reaches the IMLNs of the third and fourth space. Evidence from previous studies in locally advanced breast cancer treated by radical mastectomy or quadrantectomy show that the majority of IMLN metastases are in the second and third intercostal spaces [7]. Hence the biopsy of IMLN can be based on the anatomic location of the tumour, second intercostal space for the upper half tumours and the third for the lower half [8].

The finding of a positive IMLN in locally recurrent breast may be important in deciding to treat the patient with systemic chemotherapy as illustrated by both cases presented here, neither of which would have had chemotherapy had there been no evidence of further spread. Moreover some studies in primary breast cancer have shown a survival benefit by adding radiotherapy to the IMLN field [8, 9]. There are reports of re-irradiation of the IMLNs in some institutions showing a survival benefit but this approach is likely to be associated with a high risk of morbidity and should be viewed with caution [10, 11]. Many patients with regional lymph node recurrence have simultaneous distant metastases, emphasizing the need for a thorough staging workup when regional recurrence is detected.

Many surgeons avoid IMLN biopsy because of the perceived complexity of the procedure as well as the risk of significant complications such as bleeding, chylothorax and pneumothorax. However, studies that addressed this issue have reported only minor complications in up to 1.5% of patients undergoing the procedure [8]. In our experience IMLN biopsy only adds an additional 10 minutes to the procedure.

The value of the IMLN status as a prognostic and treatment guide tool is shown by many studies. All 3 cases that we reported here had positive IMLN and negative axilla. They had very different histories but one thing in common: the normal lymphatic pathways from the breast to the ipsilateral axilla had been disrupted by previous sentinel node biopsy or some form of axillary sampling but none had had a full dissection. These cases support the hypothesis that in cases of previous surgery to the axilla, such as recurrent breast cancer, IMLN biopsy should be a routine step irrespective of the findings on the pre-operative lymphoscintigram.
